# Improved breast cancer diagnosis using a CA15-3 capture antibody-lectin sandwich assay

**DOI:** 10.1007/s10549-025-07672-z

**Published:** 2025-03-27

**Authors:** S. Nikseresht, L. K. Shewell, C. J. Day, M. P. Jennings, H. Chittoory, A. E. McCart Reed, P. T. Simpson, S. R. Lakhani, R. Nabiee, M. Moore, R. Khanabdali, L. M. Hinch, G. E. Rice

**Affiliations:** 1 INOVIQ Ltd, Notting Hill, 23 Normanby Road, VIC Australia; 2 Institute for Glycomics, Griffith University, Gold Coast, QLD Australia; 3 Centre for Clinical Research, The University of Queensland, Brisbane, 23 Normanby Road, QLD Australia; 4 Pathology Queensland, Brisbane, Qld Australia; 5 ResearchDx, Irvine, CA USA

**Keywords:** Glycolyl neuraminic acid, SubB2M, CA15-3, Breast cancer

## Abstract

**Purpose:**

This study aims to test the hypothesis that an enzyme-linked antibody-lectin sandwich assay for a glycovariant of CA15-3 can deliver better diagnostic performance, defined by classification accuracy, sensitivity and specificity, for breast cancer compared to an existing FDA-approved CA15-3 test.

**Methods:**

A genetically engineered lectin (SubB2M) that specifically binds N-glycolylneuraminic acid (Neu5Gc) was used as a detection reagent in a CA15-3 capture antibody-lectin sandwich (neuCA15-3) assay. In a case: control cohort equivalence study the classification accuracy for the neuCA15-3 assay was determined and compared to an FDA-approved CA15-3 IVD test (Elecsys CA15-3 II, Roche Diagnostics).

**Results:**

Classification accuracy and AUC for neuCA15-3 were 81% and 0.886 ± 0.015 (standard error, *n* = 567) and for Elecsys CA15-3 II, 55% and 0.642 ± 0.023 (*n* = 558), respectively. At a threshold cut-off serum concentration of 23.6 units/ml, overall breast cancer classification accuracy of the neuCA15-3 was 81% (compared to 55% for the comparator assay, *p* < 0.001). At 95% specificity, the sensitivity of the neuCA15-3 assay was 69.5%, significantly greater than the comparator assay (11.9%, *p* < 0.001). neuCA15-3 concentrations did not vary significantly with breast cancer receptor subtype or comorbidities tested.

**Conclusions:**

The diagnostic performance of neuCA15-3 was substantially improved by specifically targeting both a CA15-3 protein epitope and a pan-cancer glycan (Neu5Gc) epitope (the specific binding target of SubB2M). The reporter signal generated depends on the colocalization of the cancer antigen protein epitope and the aberrant sialylation of the protein, thus increasing the assay specificity. The presence of multiple Neu5Gc lectin-binding sites per glycoprotein molecule increases signal generation and assay sensitivity. The inclusion of additional cancer biomarkers in a multivariate index assay format may further increase diagnostic performance for breast cancer.

**Supplementary Information:**

The online version contains supplementary material available at 10.1007/s10549-025-07672-z.

## Introduction

Oncogenic transformation is associated with the metabolic reprograming of cells, leading to a series of adaptations that promote immune evasion, resistance to apoptosis, hyperproliferation, angiogenesis, loss of contact inhibition, invasion, metastasis and a shift to aerobic glycolysis to meet energy demands and biomass synthesis [1–8]. Among these adaptations, changes in the expression and structure of cell surface glycoconjugates are crucial. Cell–surface glycoconjugates, including proteoglycans, glycoproteins and glycolipids, form the dense glycocalyx that envelop cells and play a fundamental role in cell signalling, immune modulation, cell–matrix interactions, angiogenesis and invasion [9–12].

A commonly observed change in cancer-derived glycoconjugates is the aberrant sialylation of cell surface glycoproteins [13]. Sialic acids, nine-carbon monosaccharides, typically occupy terminal positions on the oligosaccharide chains of cell surface glycoconjugates. The addition of sialic acid to these molecules significantly alters cell–cell recognition, cell signalling and environmental interactions [14–19]. While *N*-acetylneuraminic acid (Neu5Ac) is the most abundant sialic acid in humans [20], many cancers, including: melanoma; retinoblastoma; colon cancer; ovarian cancer; and breast cancer [21–26] preferentially accumulate and incorporate *N*-glycolylneuraminic acid (Neu5Gc, a hydroxylated derivative of Neu5Ac) as the terminal residue of cell surface glycans and glycoconjugates released into the peripheral circulation [26–28].

The concentration of Neu5Gc-glycoconjugates in the peripheral circulation of patients with cancer may increase up to 20-fold and is reflective of disease progression [26–30]. Serum Neu5Gc, therefore, may be an informative biomarker of cancer onset, disease progression and response to treatment [30–32]. As such, quantification of serum Neu5Gc concentrations affords an opportunity to develop diagnostics and liquid biopsy tests that are cancer-specific and more sensitive, as multiple Neu5Gc epitopes may be present per biomarker molecule. Many cancer biomarkers currently used in clinical practice are glycoproteins that may contain Neu5Gc [26–28, 30].

To progress the development of diagnostic tests that detect the presence of both cancer biomarker glycoproteins and their Neu5Gc glycosylation status, highly sensitive Neu5Gc biologicals are required. Recently, we developed a natural, Neu5Gc lectin, called SubB [33], that has been engineered (ΔS106/ΔT107) to bind Neu5Gc regardless of branched presentations (SubB2M, [26–30, 34]). The use of the SubB2M Neu5Gc-specific lectin has the potential to improve the classification accuracy of existing cancer glycoprotein in vitro diagnostic (IVD) tests. The CA15-3 biomarker test detects a shed truncated form of the transmembrane protein MUC-1. MUC-1 is composed of MUC-1-N (extracellular) that contains tandem repeated sequence motifs densely decorated with O-glycans and MUC-1-C (cytoplasmic) subunits. MUC-1 is overexpressed in many cancers [35] and, under stress, MUC-1-N is shed into the extracellular space and MUC-1-C is released into the cytoplasm where it may function in oncogenic cell signaling [36–46]. Serum concentrations of the shed ectodomain of MUC-1(*i.e.*, CA15-3), are increased in association with breast [47], ovarian [48] and pancreatic cancer [49], and non-malignant conditions, including: pregnancy [50]; coronary heart disease [51]; and inflammatory bowel disease [52]. While CA15-3 is approved for breast cancer treatment response monitoring and disease recurrence, CA15-3 serum concentrations are not consistently increased in women with early or localised breast cancer [53]. This contributes to CA15-3's low sensitivity in detecting early stage cancer [54–56]. In this study, biotinylated SubB2M was used as a detection reagent in a CA15-3 capture antibody-lectin sandwich assay. The performance of the assay was evaluated in a breast cancer case: control cohort study and compared to the Elecsys CA15-3 II test. The hypothesis to be tested was that the diagnostic performance (as defined by the classification accuracy, sensitivity and specificity) of CA15-3 to detect breast cancer is increased using a Neu5Gc-binding lectin detection reagent when compared to the Elecsys CA15-3 II assay. The Elecsys CA15-3 II test is approved by the FDA as an aid in the early detection of cancer recurrence in previously treated stage II and III breast cancer patients and for monitoring response to therapy in metastatic breast cancer.

## Materials and methods

### Recombinant expression of SubB2M

SubB2M lectin was produced by MP Biomedicals, Singapore. Briefly, a vial of SubB2M clonally selected cells (INOVIQ Ltd, Melbourne Australia, # ACL034) was thawed and cells were cultured in a sterile 500 ml conical shaking flask containing 100 ml of Luria Bertani culture medium (MP Biomedicals, Cat # 113,002,011-CF) with 100 mg/ml Ampicillin (Sigma, # A5354). The cells were incubated overnight in a 37 °C incubator with shaking at 200 rpm. Cells were then transferred and cultured in a 5 l bioreactor (BIOSTAT® Aplus Sartorius Bioreactor) with following parameters: 3 l min^−1^ compressed air, agitation speed of 200 rpm, at 37 °C, pH 7.5 and optical density was measured at 600 nm wavelength (OD). Cells were cultured for 2 h and OD measured. Once OD reached 0.6 to 0.8, a sample was collected as “uninduced” and stored at 4 °C for further analysis. Cells were then incubated in the presence of isopropyl β-d-1-thiogalactopyranoside, by the addition of 10 ml of 5 M to the bioreactor culture, to induce expression. The following day, an aliquot of the cell culture was collected and the OD measured. The sample was labelled as “induced” and stored at 4 °C for further analysis. The bioreactor cell culture was transferred into centrifuge bottles and centrifuged at 8000 rpm, 15 min, 4 °C using (Beckman Coulter Avanti J-S26 XP with Rotor JA-10). The supernatant was discarded and the cell pellets were stored at − 80 °C.

*Evaluation of Induction Process:* The “uninduced” and “induced” samples were sedimented at 10,000 rpm for 20 s. The supernatant was discarded and the cell pellet was resuspended in 200 ml of Sample Buffer (Tris (1 M, pH 6.8), 4.0 ml; SDS (20%), 5 ml; Glycerol, 5 ml; Bromophenol blue (0.1%), 300 μl) by pipetting up and down. Samples were then placed in a boiling water bath for 10 min before analysis on 12% SDS-PAGE mini-gels (Thermo Fisher) at 150 V for approximately 1.5–2 h. The gel was stained with Coomassie blue to determine induction status. If monomeric SubB2M (~ 13 kDa) was detected after induction, large-scale cell pellet preparation was proceeded for purification.

*Purification of large-scale cell pellet:* The frozen cell pellet was thawed and for every 5 g of cell pellet, 100 ml of Purification (Prelysis) buffer (50 mM Sodium Phosphate, pH 7.8) was added and cells were resuspended by vortexing. The collected cell suspension was processed in a cell disruptor (Constant Systems CF1 Cell Disruptor System, Constant Systems) at 30 kpsi at 4 °C until a clear solution was obtained. The solution was then centrifuged in 50 ml centrifuge tubes in Rotor JA-20 at 20,000 rpm for 30 min at 4 °C. The supernatant was transferred into a clean bottle and kept at 4 °C for purification.

The supernatant containing SubB2M was purified by AKTA Start (Cytiva) using His Trap HP Nickle pre-packed 5 ml columns (Cytiva). The purification was performed in a cold room (4 °C) to prevent protein degradation. Briefly, the column was washed with deionized water to remove storage buffer, and the flow-through sample was collected. Elution fractions were labelled and analysed using SDS-PAGE mini-gels. Fractions with high SubB2M content and minimal contaminants were pooled. Zeba Spin Desalting columns were used to desalt the samples. Protein concentration was determined using the Bradford assay and the purified protein was stored at − 20 °C in 25 mM sodium phosphate, 0.025% Tween-20, 50% glycerol, pH 7.8, SubB2M storage buffer).

### SubB2M biotinylation

SubB2M (1 mg) was biotinylated using an amine-reactive ester biotin-succinimidyl ester (Invitrogen™, Cat# B1606). SubB2M storage buffer was exchanged into low salt PBS (pH 8.5) and incubated with biotin-succinimidyl ester (145 µg/mg protein) at 4 °C for 2 h. The reaction was quenched by adding 1/100th volume of ethanolamine (Merck, Cat# E9508). Low salt PBS was then removed using Zeba Spin Desalting Columns, 7 K MWCO (Cat# 89,883) and protein concentration determined using Pierce BCA Protein Assay kit (Thermo Fisher Scientific, cat # 23,225). Biotinylated SubB2M was stored in 1 × PBS 0.05% Tween-20/50% glycerol (Sigma, Cat# G5516-100ML) at − 20 °C.

### Clinical samples

*Retrospective Case: Control Cohort Study:* Serum samples (1 ml) obtained from normal healthy women and women with breast cancer were purchased from a commercial biorepository (ProteoGenex Inc, Inglewood, CA, USA; see Supplementary Data Table 1A and 1B). The inclusion criteria for breast cancer samples were as follows: (a) patients with histologically confirmed breast cancer, stages I to IV; (b) no radiotherapy, chemotherapy or endocrine manipulations before the surgery; and (c) no clinical or laboratory evidence of benign diseases of the liver, pancreas, ovary or kidney. Patient staging was according to the International Union Against Cancer criteria. Histological typing was according to the WHO classification. All sample processing, storage and assays were conducted by an ISO17025-compliant Contract Research Organisation (ResearchDx, Irvine, CA, USA). Case and control samples were age matched. The median age and interquartile range (IQR) for controls was 55 (48–65) and for cases 57 (48–65), (*p* > 0.05). The median duration from sample collection to assay was not significantly different between control (median 5.2, IQR 3.0–7.1 years) and case (median 4.9, IQR 3.9–6.0 years) samples (*p* > 0.05). On receipt, 20 and 500 µl aliquots of serum were stored at − 80 °C until assayed. The concentration of CA15-3 in each serum sample was measured using both the neuCA15-3 and Roche’s Elecsys CA15-3 II assays.

A second cohort of serum from breast cancer patients was accessed from the Brisbane Breast Bank [57] (see Supplementary Data Table 2). The use of clinical samples was approved by the Human Research Ethics Committees at The University of Queensland (2005/000785) and the Royal Brisbane and Women’s Hospital (2005/022).

#### Sub2M-SubB2M sandwich capture and detection of sialylated proteins

Wells of clear NUNC MaxiSorp 96-well plates (Merck, Cat no. M9410-1CS) were coated with 100 ng/well of SubB2M in 0.05 M carbonate/bicarbonate coating buffer pH9.6. Non-bound SubB2M was removed by three washes of PBS/0.05% Tween-20 (PBS-T) before blocking with 100µL of 2% ovalbumin (Merck; Cat no. A5503-10G)/PBS-T. Sialylated proteins, bovine mucin 1, Neu5Gc-Ovalbumin and Neu5Ac-Ovalbumin with and without neuraminidase pretreatment were added across a 1:2 dilution starting at 100 µg/mL in PBS-T, with PBS-T used as the negative control for 1 h at room temperature. Neuraminidase pretreatment was carried out on 5 mg/mL stocks of the proteins with 40 units of α2-3,6,8,9 Neuraminidase (New England Biolabs) in Glycobuffer 1 for 1 h at 37 °C. Untreated protein was incubated in Glycobuffer 1 without neuraminidase for 1 h at 37 °C. Wells after incubation were washed four times with PBS-T and 100 µl/well of 100 ng/mL 64 × biotinylated SubB2M diluted in 1% ovalbumin/PBS-T was added and incubated for 1 h at room temperature. The plate was washed four times with PBS-T prior to 100 µl/well of streptavidin-HRP (1:10,000, Abcam Cat no. ab7403, stored with 50% glycerol) diluted in 1% ovalbumin/PBS-T was added and incubated for 1 h. The plate was then washed four times with PBS-T and the detection reagent was added (50 µl/well 1-step Ultra TMB-ELISA Substrate Solution ThermoFisher; Cat no. 34029) and incubated for 30 min. The reaction was stopped with 50 µl/well 1N HCl and measure absorbance at 450 nm in an Infinite 200 PRO plate reader (TECAN) with the appropriate plate settings selected and 10 flashes/well.

#### CA15-3 capture antibody-lectin sandwich assay

In this assay format, the serum breast cancer-associated biomarker is captured using a well-characterise commercially available CA15-3 monoclonal antibody. The captured protein is then detected using biotinylated SubB2M and HRP-TMB. The glycoprotein variant biomarker detected using this CA15-3 capture antibody-lectin sandwich assay is designated, in this study, as neuCA15-3 to distinguish it from CA15-3 that is detected using a pair of monoclonal antibodies (*e.g*., DF3 and 115D8). neuCA15-3 assays were performed using 96-well plates coated with anti-CA15-3 monoclonal antibody (cat #: EHMUC1, Thermo Fisher Scientific, USA). The plates were preconditioned with neuraminidase (Cat#: P0722S, New England BioLabs, USA) at 0.25 Units per µl for 2 h at 37 °C to remove antibody-associated sialic acid residues. Removal of sialic acid was monitored by the addition of 1 µg/well of both HRP conjugated *Maackia amurensis* agglutinin (MAA) and *Sambucus nigra* agglutinin (SNA-I) (EY Laboratories MAA H-7801–1/SNA-I H-6802–1) in the place of SubB2M in a column of the plate. Plates were then blocked using 300 µl /well of Superblock T20 (Cat#:37,516 Thermo Fisher Scientific, USA) for 16 h at 4 °C. The plate was washed (3 × 100 µl) using a plate washer with 1× PBST (Cat#:28,352 Thermo Fisher Scientific, USA), serum samples were diluted 16 fold with 1% Ovalbumin in PBST (Ovalbumin Cat#: A5503 Sigma Aldrich, USA) and 100 µl was added to each well and incubated for 120 min at 22 °C. Wells were washed (4 × 100 µl) to remove non-antibody-bound material and then incubated with 100 µl/well biotinylated SubB2M at 250 ng/ml for 60 min at 22 °C. Wells were washed (4 × 100 µl) and then incubated with Streptavidin-HR diluted 10,000 × ,Cat#:7403 Abcam, USA) for 60 min at 22 °C and then washed (3 × 100 µl). 50 µl/well 1-Step Ultra TMB-ELISA Substrate Solution (Cat#:34,029 Thermo Fisher Scientific, USA) was added for 12 min at RT and the reaction was stopped by the addition of 50 µl Stop Solution (Cat#SS04 Thermo Fisher Scientific, USA) and absorbance measured at 450 nm. CA15-3 calibrator (Cat # MBS5303614, MyBiosource, San Diego, CA, USA) was serially diluted to 250, 160, 80, 40, 20, 7.5, 2.5 and 0 units/ml in triplicates and quality control samples were included in duplicate on each plate. CA15-3 concentrations were interpolated from 4PL regression of the calibrator dilution series (see Supplementary Data Fig. 1). The effects of different CA15-3 capture antibodies on signal generation are summaries in Supplementary Data Fig. 2).

**Fig. 1:**
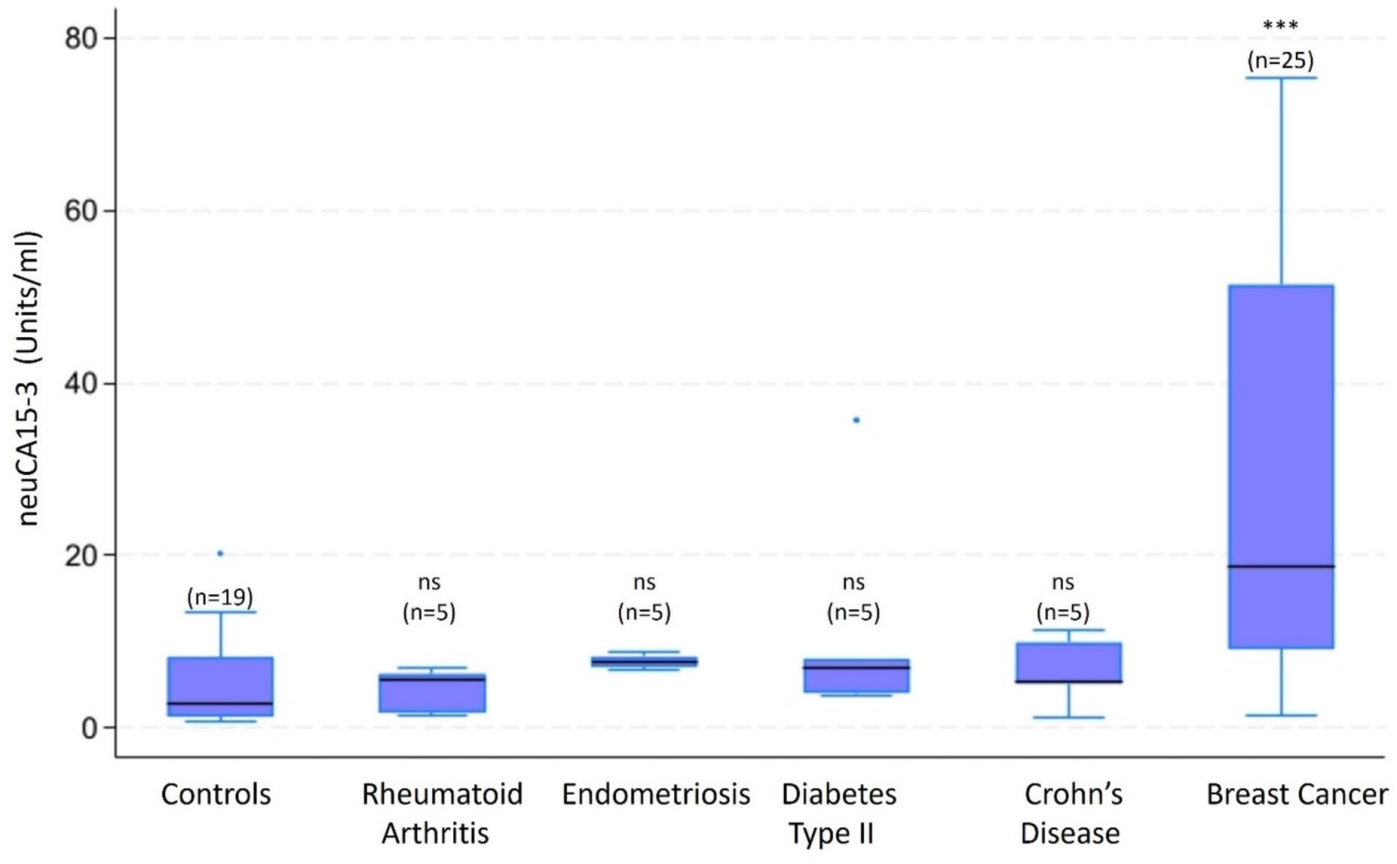
Disease-specific variations in neuCA15-3 serum concentrations are presented as the median (black line), interquartile range (box) and minimum and maximum values (lower and upper whiskers). The overall variation in neuCA15-3 concentrations was assessed using a Kruskal–Wallis one way analysis of variance. Pairwise comparisons to the controls were assessed using Dunn’s tests (with Bonferroni correction for multiple hypothesis testing). No statistically significant difference between median concentrations was observed for control and inflammatory disease (ns). The median neuCA15-3 concentration for Breast Cancer was significantly greater than that of controls ***p < 0.001)

**Fig. 2:**
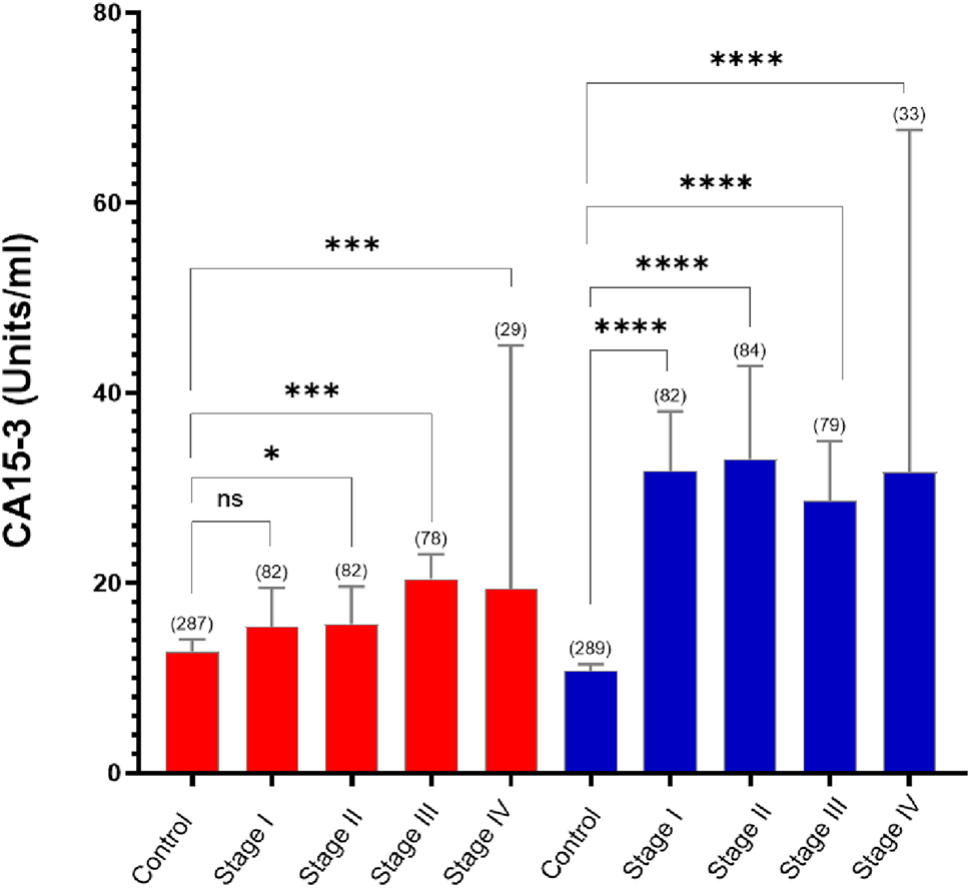
Stage-specific variation in CA15-3 serum concentrations measured using neuCA15-3 (blue bars) and Elecsys CA15-3 II (red bars) assays are presented as the median and 95% confidence intervals (*n*). For each assay, the overall variation in CA15-3 concentrations was assessed using a Kruskal–Wallis one way analysis of variance (neuCA15-3, *p* = 0.0001; Elecsys II CA15-3, *p* = 0.0001). Pairwise comparisons of the stage-specific variation in CA15-3 were assessed using Dunn’s tests (with Bonferroni correction for multiple hypothesis testing). At all stages of breast cancer, the median neuCA15-3 concentration was significantly greater than that of controls. No statistically significant difference between Elecsys II CA15-3 median concentrations was observed for control and Stage I breast cancer. At all other stages, the median Elecsys II CA15-3 concentration was significantly greater than that of controls. (*p < 0.05; ***p < 0.001; ****p < 0.0001)

#### Disease Specificity of the CA15-3 capture antibody-lectin sandwich assay

The potential confounding effect of other clinical conditions (including inflammatory disease) on the measurement of serum concentrations of CA15-3 using the capture antibody-lectin sandwich assay was assessed using serum samples obtained from patients with Crohn’s Disease (*n* = 5), Diabetes Type II (*n* = 5); Rheumatoid Arthritis and Endometriosis (*n* = 5). Serum samples (1 ml) obtained from normal healthy women, women with inflammatory diseases and women with breast cancer were purchased from a commercial biorepository (ProteoGenex Inc, Inglewood, CA, USA). For direct comparison, serum obtained from healthy controls (*n* = 19) and patients with diagnosed breast cancer (*n* = 25) were measured in the same assay.

#### Elecsys CA15-3 II assay

CA15-3 serum concentrations were quantified using the Elecsys CA15-3 II (Cat#: 07027001190 Roche, USA) on a Cobas 8000 Modular Analyzer instrument and were performed under a commercial contract with Pacific Medical Laboratory (CLIA ID Number: 05D0692370), Irvine CA, USA. Assays were conducted according to manufacturer’s instructions. The assay has a reported range of 1–300 units/mL and a sensitivity of 30 units/ml.

#### Statistical analysis

CA15-3 concentration data homogeneity of variance was assessed using Shapiro–Wilk tests [58] and found to significantly deviate from a normal distribution. Statistical analyses, therefore, were performed using nonparametric tests. CA15-3 concentrations are represented as the median value with the interquartile range (IQR) or 95% confidence intervals, as indicated. Between-group differences were assessed using two-sample Mann–Whitney* U* tests [59] or Kruskal–Wallis tests and post-hoc Dunn’s tests for pairwise comparisons [60]. The classification accuracy and Receiver Operating Characteristics for both assays was assessed using binary logistic regression analysis (Stata Ver 18, StataCorp College Station, TX, USA and the pROC package in R).

## Results

### Confirmation of SubB2M detection of Neu5Gc decorated proteins

SubB2M has been shown to be specific for Neu5Gc in glycoconjugate arrays and SPR analysis [25–28, 32], however, limited assays have been performed to confirm its specificity in enzyme-linked antibody-lectin sandwich assays. To test its specificity for Neu5Gc in ELISA-like assays, both as a capture and detection reagent, a SubB2M–SubB2M sandwich assay was performed using bovine Mucin 1 and ovalbumin chemically modified with Neu5Ac or Neu5Gc. The detection of proteins was tested with and without neuraminidase pre-treatment. It was found that binding to bovine Mucin 1 and Neu5Gc–Ovalbumin was sialic acid dependent, and Neu5Ac–Ovalbumin was not recognised (Supplementary Data Fig. 3).

**Fig. 3:**
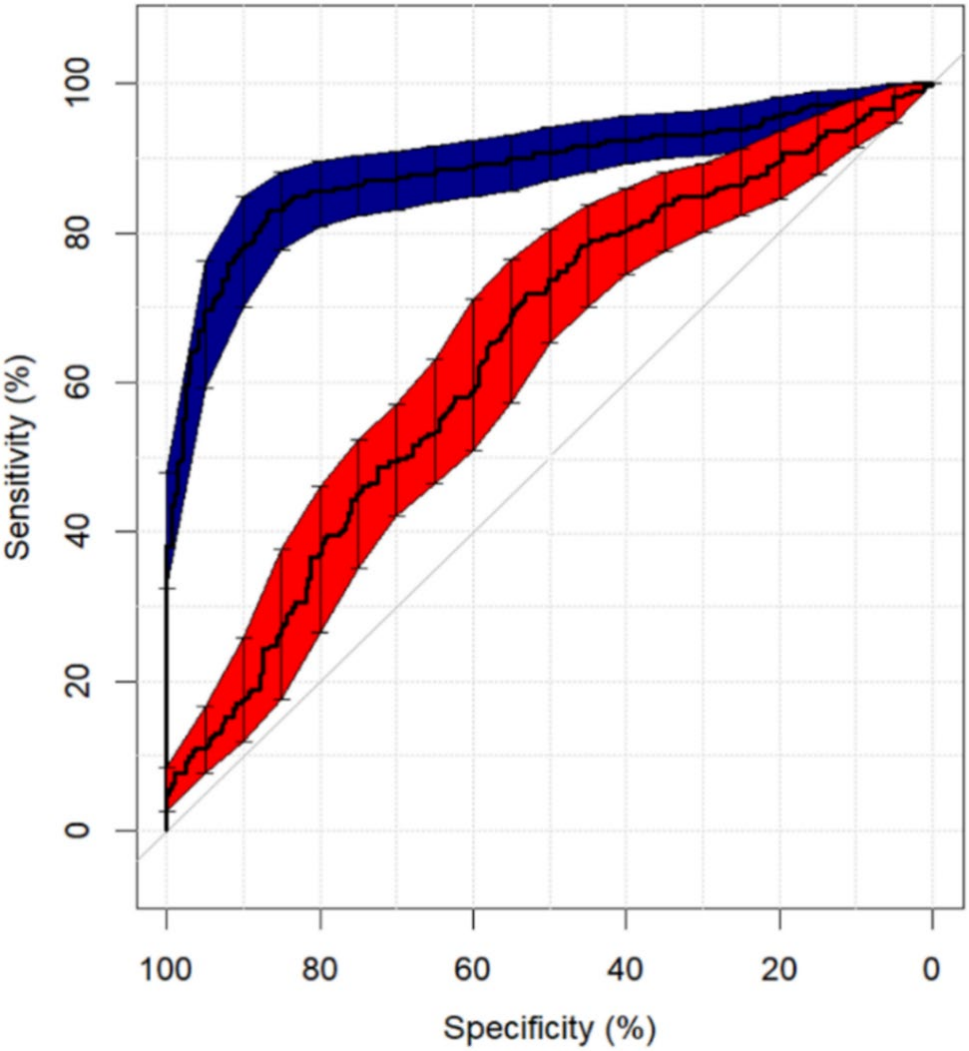
Receiver Operating Characteristic Curves for neuCA15-3 (blue) and Elecsys CA15-3 II (red) assays. The AUCs for neuCA15-3 and Elecsys CA15-3 II assay were 0.886 ± 0.015 (standard error) and 0.642 ± 0.023. Sensitivity 95% confidence intervals are likelihood ratio reported for each assay at their respective cut-off thresholds

### Murine capture antibody pre-treatment

Murine antibodies, including IgGs, can be decorated with Neu5Gc-containing glycans [61]. The presence of antibody-associated Neu5Gc (the binding target of SubB2M), therefore, may contribute to non-specific binding and reduced assay sensitivity. SubB2M reacts with a range of mouse CA15-3 antibodies including: 115D8 (Thermo Fisher Scientific, Cat No. MA1-35,039), 11FD8 (Thermo Fisher Scientific, Cat No. TA600445) and the capture antibody from the Human/MUC-1 ELISA kit (Thermo Fisher Scientific, Cat No. EHMUC1). To eliminate any confounding effects of antibody-associated Neu5Gc, the capture antibody used in all neuCA15-3 assays using SubB2M as a detection reagent was pretreated with α2–3,6,8,9 neuraminidase. The effect of neuraminidase pre–treatment of mouse monoclonal antibodies on SubB2M binding is presented in Supplementary Data Fig. 2. The pre-treatment of two CA15-3 murine monoclonal antibodies with α2–3,6,8,9 neuraminidase significantly reduced the non-specific binding of SubB2M (*p*< 0.001). In all subsequent assays, using SubB2M as a detection reagent, the monoclonal capture antibody was pretreated with α2–3,6,8,9 neuraminidase.

### Neu5Gc CA15-3 analytical validation

The immune-lectin sandwich assay for CA15-3 reported in the manuscript was designed to detect immunoreactive CA15-3 captured by monoclonal antibody DF3 and detected by the presence of neu5Gc epitopes on the DF3-captured CA15-3. The sequence of the DF3 epitope is DTRPAPGS. This sequence is part of the heavily glycosylated extracellular domain of the transmembrane protein MUC-1, which is often overexpressed and abnormally glycosylated in various cancers [26–28, 30, 62]. The immune-lectin sandwich assay is referred to as neuCA15-3 to differentiate it from the Elecsys CA15-3 II sandwich monoclonal antibody assay.

Analytical validation of the neuCA15-3 assay was performed to the requirements of the Clinical and Laboratory Standards Institute’s Evaluation of Detection Capability for Clinical Laboratory Measurement Procedures EP17-A2 standard. Assay performance metrics were estimated from 13 independent assays conducted over 4 days. A summary of relevant assay performance measures is presented in Table 1. The limits of quantitation were 1–250 units/ml with an overall inter-assay coefficient of variation of 10%.

**Table 1:** neuCA15-3 Analytical validation

Limits of detection	
Limit of blank (matrix) Units/ml	0.33
Limit of blank (serum) Units^/^ml	0.52
Limit of detection (serum blank) Units/ml	0.50
Limit of detection (low positive sample blank) Units/ml	0.87
Signal to noise ratio	20.50
*Recovery* [Tumor control samples Fujirebio]	
Low positive (13 Units/ml)	0.91
High positive (157 Units/ml)	1.07
median recovery rate	0.99
median relative error rate	0.08
Precision	
Inter-assay coefficient of variation (%)	
Low positive sample (5.6 Unit/ml)	13%
Medium Positive sample (74.8 Units/ml)	12%
High positive (203.8 Units/ml)	3%
Overall coefficient of variation	10%
Intra-assay coefficient of variation	1.4%
*Linearity* (*r*^2^ estimate)	0.97

Assay performance metrics were estimated from 13 independent assays. Limits of detection were estimated using CA15-3 calibrator (MyBiosource Cat # MBS5303614, isolated from human ascites fluid). Tumor Marker Controls (Cat # 108-20W Fujirebio, Tokyo, Japan) were used to estimate recovery and assay precision

### Disease Specificity of the neuCA15-3 capture antibody-lectin sandwich assay

Variation in neuCA15-3 serum concentrations with rheumatoid arthritis, endometriosis, diabetes type II and Crohn’s disease was assessed using an independent sample set (*n* = 64, Fig. 1). No significant difference in neuCA15-3 concentrations between inflammatory diseases and healthy controls were observed, as assessed using Kruskal–Wallis test (*p* > 0.05). Serum neuCA15-3 concentration in breast cancer was significantly greater than controls (*p* < 0.001).

### Retrospective case: control cohort study

To benchmark the neuCA15-3 against the routine FDA-approved CA15-3 test, we made a direct comparison of the diagnostic performance of both tests using serum samples obtained from healthy women and women already diagnosed with breast cancer using both assays. Breast cancer serum samples were collected after diagnosis and before treatment. CA15-3 concentrations were not normally distributed (Shapiro-Wilks normality test, *p* < 0.001), therefore, non-parametric statistics were used in subsequent analyses. In control samples, CA15-3 concentrations were significantly lower when measured using the neuCA15-3 assay (median = 10.7 (IQR 4.8–15.1) Units/ml, *n* = 289) compared to the Elecsys CA15-3 II assay (median = 12.8 (8.9–19.4) Units/ml, *n* = 287) (*p* < 0.0001, Mann–Whitney two-tailed test). In breast cancer samples, the median neuCA15-3 concentration (31.4, 22.2–50.5 Units/ml, *n* = 291) was approximately 1.8-fold higher than that measured by the Elecsys CA15-3 II assay (median = 17.3, 12.3–25.8 Units/ml, *n* = 287) (Fig. 2, *p* < 0.0001).

Over all samples, CA15-3 concentrations measured by the two assays were significantly correlated, as determined by Spearman’s rank correlation (rho = 0.21, *p* < 0.001, *n* = 557 paired observations). The correlation between assay CA15-3 concentrations, however, varied significantly with disease stage; with no significant correlation observed between the two assays in Stage I samples (rho = 0.148, *p* = 0.263, *n* = 27) but a strong correlation observed in Stage IV samples (rho = 0.657, *p* = 0.002, *n* = 29, Supplementary Table 3).

The stage-specific variation in serum CA15-3 concentrations is presented in Fig. 2. neuCA15-3 serum concentration from breast cancer patients were 1.5 to 2-fold higher than those reported in the Elecsys CA15-3 II assay, and at all stages of breast cancer were significantly greater than those observed in normal healthy controls, as assessed by Kruskal–Wallis test and post-hoc Dunn’s Pairwise Comparison test (using a Bonferroni corrected for multiple hypothesis testing)

The association between comorbidities, including hypertension *n* = 35; gastric and colon inflammatory diseases *n* = 14; coronary artery diseases *n* = 13; obesity *n* = 15 between neuCA15-3 serum concentrations and disease status was assessed by multivariate regression analysis. No statistically significant associations were identified (Supplementary Data Table 5).

**Receiver Operating Characteristic Curves:** The classification accuracy of the two CA15-3 assays was compared using Receiver Operating Characteristic (ROC) curve analysis. The accuracy of the two tests to correctly classify breast cancer samples is summarised in Fig. 3. The cut-off threshold concentration for each assay was defined as the 95% percentile concentration of the normal healthy controls and were, for the neu5Gc CA15-3 and Elecsys CA15-3 II assays, 23.6 and 34.7 Units/ml, respectively. Overall classification accuracy and area under the curve (AUC) was 80.8% and 0.886 ± 0.015 for neuCA15-3 (*n* = 567), and 54.7% and 0.642 ± 0.023 for the comparator (*n* = 558), *p* < 0.0001).

**Fig. 4:**
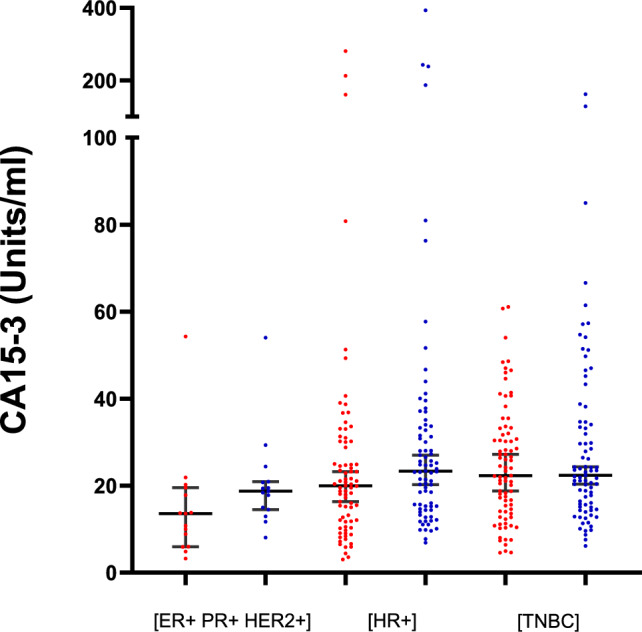
Variation in CA15-3 serum concentrations by receptor subtypes (ER + PR + HER2 + (*n* = 15); HR + (*n* = 78); and TNBC (*n* = 81)) measured using the neuCA15-3 (blue) and Elecsys II (red) CA15-3 assays. Data are presented as a scatter diagram displaying the median CA15-3 concentration with 95% confidence intervals. CA15-3 serum concentrations did not vary significantly with breast cancer receptor subtype

***Breast cancer receptor status****.* The variation in serum CA15-3 concentrations, by breast cancer receptor status (HER2 + ; HR + and TNBC), was evaluated in an additional 174 breast cancer serum samples (see Supplementary Table 4) and is presented in Fig. 4. No statistically significant differences in neuCA15-3 concentrations between receptor subtypes was identified (Kruskal–Wallis test, *p* = 0.21). Elecsys CA15-3 II concentrations significantly varied between receptor subtype group (*p* = 0.006), with ER + HR + HER2 + < HR + = TNBC (*p* = 0.03 and 0.003, respectively, as assessed by post-hoc Dunn's test).

## Discussion

Most serum biomarkers of cancer, including CA15-3, are based on detection of heavily glycosylated proteins that can incorporate *N*-glycolylneuraminic acid as terminal sialic acid residues on oligosaccharide chains. The detection of aberrant sialoglycans on glycoproteins produced and shed from cancer cells, therefore, offers an additional dimension to enhance specificity and sensitivity of cancer biomarker assays [63–65]. The preferential incorporation of Neu5Gc into cancer-derived glycoconjugates may distinguish them from those produced by normal healthy tissues and in various benign conditions [29]. The targeted detection of Neu5Gc-containing glycoconjugates, here CA15-3, using appropriate lectins or antibodies, therefore, may improve the specificity of existing cancer biomarker immunoassays, as demonstrated with the neuCA15-3 assay. Moreover, the sensitivity of glycoprotein biomarker assays may be enhanced by targeting the many repeated glycan epitopes, such as Neu5Gc capped glycans on cancer-derived glycoconjugates, using lectins (and IgM antibodies) for high affinity and avidity binding detection. Under normal conditions, Neu5Gc is found at extremely low concentrations in healthy human tissues [66] and serum [20], where concentrations (3.3 pg Neu5Gc/μl serum) are approximately 10,000 times lower than Neu5Ac (the most abundant form of neuraminic acid in humans). The source of Neu5Gc that is incorporated into cancer biomarkers remains the subject of contemporary interest and debate [22, 30, 31, 67–69]. Humans are unable to convert Neu5Ac into Neu5Gc due to a genetic mutation in the *CMAH* gene, that results in an inactive cytidine monophosphate *N*-acetylneuraminic acid hydroxylase. Both dietary intake [68, 70–72] and non-CMAH *de novo* endogenous synthesis [30, 73] have been proposed as sources of Neu5Gc.

In this study, a novel lectin (SubB2M), genetically engineered to specifically bind Neu5Gc containing glycoproteins, was used as the detection reagent in a CA15-3 capture antibody sandwich enzyme-linked lectin-binding assay and the diagnostic performance of the assay was compared with an FDA-approved CA15-3 IVD assay (Elecsys CA15-3 II, Roche Diagnostics). The data obtained establish that the neuCA15-3 assay is analytically robust and reproducible, and displays high classification accuracy for all stages and receptor subtypes of breast cancer. Furthermore, the neuCA15-3 test was not confounded by inflammatory disease (tested herein). The assay was analytically validated to CLIS standards and reported fit-for-purpose performance characteristics.

The diagnostic performance (as defined by the classification accuracy, sensitivity and specificity) of the neuCA15-3 assay was assessed in a breast cancer case: control cohort study. The median neuCA15-3 serum concentrations in healthy women (10.7 units/ml) were significantly lower than that reported for Elecsys CA15-3 II assay. This is consistent with the former assay only detecting CA15-3 that contains Neu5Gc. The detection of low concentrations of Neu5Gc-CA15-3 in normal healthy women may be the result of increased sensitivity of the neuCA15-3 assay; dietary intake of Neu5Gc (e.g., dairy and red meat products); de novo non-enzymatic formation of Neu5Gc; and/or low-level cross-reactivity with residual Neu5Ac.

The observed improvement in the classification accuracy using the neuCA15-3 assay (81 vs 55%, for the Elecsys CA15-3 II assay) is consistent with more specific targeting of cancer-derived Neu5Gc-CA15-3 and increased assay sensitivity. In support of the latter, Choi et al., previously reported that the use of a non-specific lectin (concanavalin A) as a detection reagent also increased CA15-3 assay sensitivity [74].

To date, there has been limited success in translating antibody-lectin sandwich assays into routine clinical practice [16, 17]. One of the main issues has been the non-specific binding of lectin-based detection reagents to sialic acid residues present on coating or capture antibodies and/or present in blocking agents that contain mammalian glycoproteins. In this study, the use of a Neu5Gc-specific lectin, neuraminidase pretreatment of capture antibodies containing Neu5Gc and the use of avian protein blocking agent (ovalbumin that does not contain Neu5Gc) significantly reduced this source of non-specific binding. The modifications of the CA15-3 assay described herein may improve the translation of lectin-binding immunoassays into routine clinical practice.

CA15-3 assays have been approved by the FDA for the measurement of CA15-3 antigen to aid in the management of previously diagnosed breast cancer patients, and in conjunction with other clinical and diagnostic procedures, as an aid in the early detection of recurrence in previously treated Stage II and III breast cancer patients for monitoring response to therapy in metastatic breast cancer patients. Analysis of the classification accuracy of the neuCA15-3 assay by tumour stage is indicative that this assay may also have utility as a screening test for early-stage breast cancer (*i.e.*, Stage I & II). As a single standalone biomarker, the sensitivity of neuCA15-3 at 95% specificity was 69% for stage I & II and compares favourably to mammography. Combining neuCA15-3 with other Neu5Gc-containing cancer biomarkers in a multivariate index assay may further increase diagnostic performance.

***Caveats and limitations***. This study establishes the equivalence of the neuCA15-3 test with the FDA-approved Elecsys CA15-3 II test in classifying breast cancer and cancer-free control serum samples. Furthermore, superiority of the neuCA15-3 test was established. The classification accuracy of the test in a case: control cohort study, however, cannot be extended to inform its performance in an asymptomatic population. The current study was not designed to evaluate the performance of the neuCA15-3 assay in the context of a screening test for asymptomatic populations and further prospective cohort studies would be required to robustly evaluate its suitability as a standalone or multimarker breast cancer screening test or as an adjunct to indeterminate imaging.

***Conclusion*** The use of a genetically engineered lectin detection reagent (SubB2M) and neuraminidase treatment of the CA15-3 capture monoclonal antibody substantially improves the classification accuracy of the cancer biomarker CA15-3. The improved assay may better inform the clinical management of patients previously diagnosed with breast cancer and may have potential in a multimarker screening modality for early-stage disease in combination with other breast cancer biomarkers. The improvement in diagnostic assay performance observed for CA15-3 may also be applicable for other glycoprotein cancer biomarkers that express Neu5Gc, such as CA125, prostate-specific antigen, human epididymis protein 4 and carcinoembryonic antigen. 

## Supplementary Information

Below is the link to the electronic supplementary material.

Supplementary file1 (DOCX 239 KB)

## Data Availability

No datasets were generated or analysed during the current study.
