# Supplementary material for: Improved breast cancer diagnosis using a CA15-3 capture antibody-lectin sandwich assay
Source: Breast Cancer Res Treat. 2025 Mar 27;211(3):605–15. doi: 10.1007/s10549-025-07672-z (PMC12031999; doi:10.1007/s10549-025-07672-z)

# Supplementary Data

Improved breast cancer diagnosis using a CA15-3 capture antibody-lectin sandwich assay

Nikseresht S ^1^, Shewell LK ^2^, Day CJ ^2^, Jennings MP ^2^, Chittoory, H^3^, McCart Reed, AE^3^, Simpson, PT^3^, Lakhani, SR ^3,4,^ Nabiee R^5^, Moore M^5^, Khanabdali R^1^, Hinch LM^1^ and Rice GE ^1,3#^

**Supplementary Data Table 1A** Clinical Metrics - Retrospective case: control sample cohort. Age, Body Mass Index (BMI) and the duration of serum sample storage are presented as the median value, interquartile range (IQR) and n. Variation between case and control samples were assessed by Kruskal-Wallis rank tests (and Dunn’s tests for pairwise comparison, were appropriate). No significant between group variation was identified for Age (p=0.22) or duration of sample storage (p=0.30). BMI was significantly greater in healthy subjects when compared to breast cancer patients (p < 0.01). BMI did not vary significantly with stage of breast cancer (p=0.64). To define the contribution of BMI to the observed variation in CA15-3 concentrations, Spearman’s rank correlations were performed to estimate the coefficient of determination (*i.e.,* rho^2^). Rho^2^ for Elecsys CA15-3 II and neuCA15-3 were 0.017 and 0.023, respectively. Variation in BMI thus may explain < 2.5% of the observed variation in CA15-3 concentrations.

| Clinical Status | Age | IQR | n | BMI | IQR | n | Duration of Storage (years) | IQR | n |
| --- | --- | --- | --- | --- | --- | --- | --- | --- | --- |
| Healthy | 55 | 48-64 | 289 | 27.00 | 25-28 | 239 | 5.20 | 2.9-7.1 | 242 |
| S-I | 59 | 52-66 | 82 | 25.20 | 23-27 | 67 | 5.70 | 3.9-6.4 | 75 |
| S-II | 57 | 50-65 | 84 | 25.50 | 23-27 | 71 | 5.20 | 4.0-6.0 | 72 |
| S-III | 56 | 47-66 | 80 | 25.40 | 23-27 | 64 | 4.60 | 3,3-5.8 | 74 |
| S-IV | 53 | 47-64 | 33 | 25.20 | 23-28 | 24 | 4.50 | 4.0-5.5 | 26 |
| All samples | 56 | 48-64 | 568 | 26.10 | 24-28 | 465 | 5.10 | 3.5-6.5 | 489 |

**Supplementary Data Table 1 B:** Breast Cancer Classification summarising Histological Diagnosis, Stage, Grade and Tumor, Node, Metastasis staging for Infiltrating Ductal Carcinoma, Infiltrating Lobular Carcinoma and Other Breast cancers

**Supplementary Data Table 2: Clinical Metrics** Breast cancer receptor subtype clinical sample cohort. Samples were stratified into three receptor subtype groups for analysis (HER2+, HR+ and TNBB). The representation of tumor Type, Grade, Size and Lymph node engagement are summarised as n; percent.

|  |  |  | Group (n;%) | | |
| --- | --- | --- | --- | --- | --- |
| Features | | n | HER2+ (n=43) | HR+ (n=50) | TNBC (n=43) |
| Type | ER pos | 50 | 0; 0% | 50;100% | 0; 0% |
|  | PR pos | 50 | 0; 0% | 46;92% | 0; 0% |
|  | HER2+ | 45 | 45;100% | 0/0% | 0; 0% |
| Grade | Grade 1 | 2 | 0; 0% | 2; 100% | 0; 0% |
|  | Grade 2 | 64 | 11;17% | 41;64% | 12;19% |
|  | Grade 3 | 68 | 30;44% | 7;10% | 31;46% |
| Size | T1 | 54 | 16;30% | 26;48% | 12;22% |
|  | T2 | 60 | 20; 33% | 18;30% | 22;37% |
|  | T3 | 19 | 7;37% | 5;26% | 7;37% |
| Lymph nodes | Pos | 40 | 15;38% | 12;30% | 13;13% |
|  | Neg | 98 | 30;31% | 38;39% | 30;30% |
| **Age years** (range; median) | | | 37-93; 54 | 25-89; 62 | 29-86; 57 |

**Supplementary Data Table 3** Spearman’s correlation analysis of CA15-3 concentrations measured using the neuCA15-3 and Elecsys CA15-3 II assays. Data represent correlation coefficient (rho), p-value and sample size (n) for control and breast cancer (by Stage) samples, and for all samples (Overall).

| Stage | rho | p | n |
| --- | --- | --- | --- |
| Control | -0.18 | 0.003 | 287 |
| Stage I | 0.138 | 0.215 | 82 |
| Stage II | 0.263 | 0.017 | 82 |
| Stage III | 0.134 | 0.130 | 77 |
| Stage IV | 0.657 | 0.0002 | 29 |
| Overall | 0.205 | <0.001 | 557 |

**Supplementary Data Table 4**: Breast cancer receptor subtype sample cohort obtained from The Brisbane Biobank. The distribution of breast cancer receptor subtypes, grade, size and lymph node status are presented as number per group and percentage of cohort.

| Features |  | n | HER2+ (n=43) | HR+ (n=50) | TNBC (n=43) |
| --- | --- | --- | --- | --- | --- |
| Type | ER pos | 50 | 0; 0% | 50;100% | 0; 0% |
|  | PR pos | 50 | 0; 0% | 46;92% | 0; 0% |
|  | HER2+ | 45 | 45;100% | 0/0% | 0; 0% |
| Grade | Grade 1 | 2 | 0; 0% | 2; 100% | 0; 0% |
|  | Grade 2 | 64 | 11;17% | 41;64% | 12;19% |
|  | Grade 3 | 68 | 30;44% | 7;10% | 31;46% |
| Size | T1 | 54 | 16;30% | 26;48% | 12;22% |
|  | T2 | 60 | 20; 33% | 18;30% | 22;37% |
|  | T3 | 19 | 7;37% | 5;26% | 7;37% |
| Lymph nodes | Pos | 40 | 15;38% | 12;30% | 13;13% |
|  | Neg | 98 | 30;31% | 38;39% | 30;30% |
| Age years range: median |  |  | 37-93; 54 | 25-89; 62 | 29-86; 57 |

**Supplementary Data Table 5**: A multivariate regression analysis (neuCA15-3 serum concentration and disease status x comorbidities) was performed to identify potential confounding effects of comorbidities on neuCA15-3 serum concentrations. Comorbidities included in the analysis were: hypertension n= 35; gastric and colon inflammatory diseases n= 14; coronary artery diseases n= 13; obesity n= 15 and other n= 13. No statistically significant associations between neuCA15-3 or disease status and comorbidities were identified.

# Supplementary Figures

**Supplementary Data Figure 1**: neuCA15-3 standard curve. Data were obtained from 13 independent assays over 4 days. Data represent mean absorbance ± standard deviation.


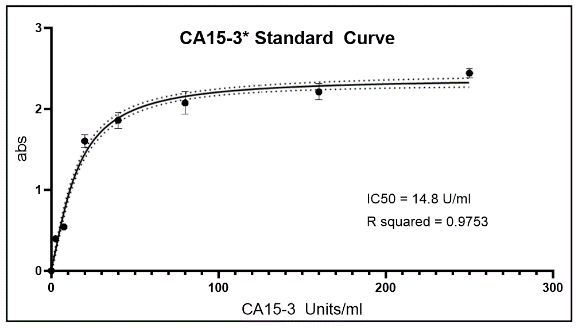


**Supplementary Data Figure** 2 Effect of different CA15-3 capture antibodies on signal geneation in the antibody-lectin sandwich assay. CA15-3 callibrator curves were performed using different capture (coating) antibodies (DF3, 115D8 and 6A4). Smilar calibration curvers were observed for the ThermoFisher. 115D8 and DF3 monoclonal antibodies.

**Supplementary Data Figure** 3: Effect of neuraminidase pre-treatment on mouse monoclonal antibody binding of biotinylated SubB2M.

**Panel A**: Analysis of SubB2M recognition of heavily sialylated proteins with and without neuraminidase treatment as per Material and Methods section. Bovine Mucin and Neu5Gc decorated ovalbumin are detected by SubB2M only in the absence of neuraminidase, while Neu5Ac ovalbumin is neither detected nor affected by neuraminidase treatment. Data represent mean absorbance ± SD (n=2).

**Panel B & C:**  Data are presented for two different mouse monoclonal (mAb) antibodies 115D8 (B) and 11FD8 (C). 96 well plates were coated with increasing concentrations of mAb, incubated and washed as per Material and Methods section. Wells were then treated with either neuraminidase (Cat#: P0722S, New England BioLabs, USA) at 0.25 Units per µl or coating buffer for 2 h at 37^o^C. Wells were washed and incubated with 500 ng/ml biotinylated SubB2M for 60 min at 22^o^C. Signal was developed by the addition of streptavidin-HRP and TMB. The reaction was stopped and absorbance at 450 nm wavelength was recorded. Data represent mean absorbance ± SD (n=3). HRP-conjugated lectins *Maackia amurensis agglutinin* (MAA) and *Sambucus nigra agglutinin* (SNA) were used as controls to confirm the activity of neuraminidase. These sialic acid binding lectins display a mouse mAb dose-dependent response in the absence of neuraminidase. Removal of sialic acid resides from the mAb by pre-treatment with neuraminidase abolishes the HRP-MMA/SNA signal.

Data are presented for two different monoclonal (mAb) capture antibodies (11FD8 – top panel and 115D8 -lower panel). 96 well plates were coated with increasing concentrations of capture mAb, incubated and washed as per Material and Methods section. Wells were then treated with either neuraminidase (Cat#: P0722S, New England BioLabs, USA) at 0.25 Units per µl or coating buffer for 2 h at 37^o^C. Wells were washed and incubated with 500 ng/ml biotinylated SubB2M for 60 min at 22^o^C. Signal was developed by the addition of streptavidin-HRP and TMB. The reaction was stopped and absorbance at 450 nm wavelength was recorded. Data represent mean absorbance ± SD (n=3).


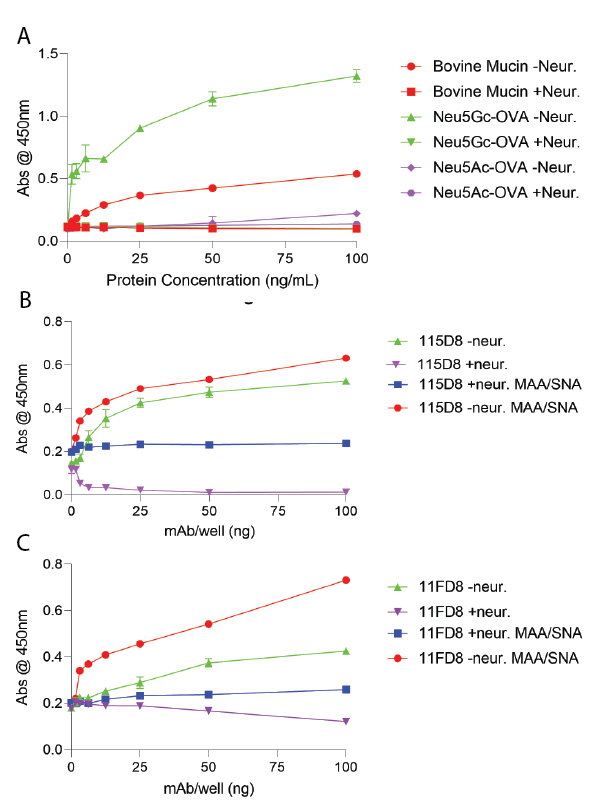

Supplement: Supplementary file 1 — Supplementary file1 (DOCX 239 KB) [file 10549_2025_7672_MOESM1_ESM.docx]
